# Analysis of sensitivity and specificity: precise recognition of neutrophils during regeneration of contused skeletal muscle in rats

**DOI:** 10.1080/20961790.2020.1713432

**Published:** 2020-03-19

**Authors:** Jiajia Niu, Guoshuai An, Zhen Gu, Peng Li, Qiqing Liu, Rufeng Bai, Junhong Sun, Qiuxiang Du

**Affiliations:** a School of Forensic Medicine, Shanxi Medical University, Jinzhong, China; b Criminal Investigation Brigade, Zhuji Public Security Bureau, Zhuji, China; c 2011 Cooperative Innovation Center of Judicial Civilization, Beijing, China; d Key Laboratory of Evidence Science, China University of Political Science and Law, Ministry of Education, Beijing, China

**Keywords:** Forensic sciences, forensic pathology, immunohistochemistry, TissueFAXS 200 digital pathological analysis system, parameter optimization, cut-off value of staining area, neutrophil staining area

## Abstract

In this report, we applied the TissueFAXS 200 digital pathological analysis system to rapidly and accurately identify neutrophils during regeneration of contused skeletal muscle, and to provide information for follow-up studies on neutrophils to estimate wound age. Rat injury model was established, and skeletal muscle samples were obtained from the control group and contusion groups at 1, 1.5, 2, 3, 4, and 6 h, as well as at 1, 3, 5, and 15 d post-injury (*n* = 5 per group). The expression of nuclei and neutrophils was detected by hematoxylin and eosin (HE) staining and immunohistochemical (IHC) staining. A total of 20 injury site areas of 0.25 mm^2^ (0.5 mm × 0.5 mm) were then randomly selected at all time points. A TissueFAXS 200 digital pathological analysis system was used to identify the positive and negative numbers. Knowledge of five professional medical workers were considered the gold standard to measure the false positive rate (FPR), false negative rate (FNR), sensitivity, specificity, and area under the curve (AUC) of receiver operating characteristic (ROC) curves. As a result, with a staining area of neutrophils from 8 µm^2^ to 15 µm^2^, the FPR was 4.28%–12.14%, the FNR was 12.42%–64.08%, the sensitivity was 35.92%–87.58%, the specificity was 87.86%–95.72%, the Youden index was 0.316–0.754, the accuracy was 82.80%–88.30%, and the AUC was 0.771–0.826. The AUC was largest when the cut-off value of the staining area was 12 µm^2^. Our results show that this software-based method is more accurate than the human eye in evaluating neutrophil infiltration. Based on the sensitivity and specificity, neutrophils can be accurately identified during regeneration of contused skeletal muscle. The TissueFAXS 200 digital pathological analysis system can also be used to optimize conditions for different cell types under various injury conditions to determine the optimal cut-off value of the staining area and provide optimal conditions for further study. Furthermore, it will provide evidence for forensic pathology cases.

## Introduction

Acute skeletal muscle injury, particularly mechanical injury, is one of the most common injuries in forensic pathology, with frequencies of 10%–50% [1,2]. After acute skeletal muscle injury, the center of the injury shows infiltration by inflammatory cells such as neutrophils and macrophages [3]. Neutrophils play important roles in the early stages of inflammation after various tissue injuries. It has been reported that neutrophils may be a useful marker for wound aging in the field of forensic medicine [4,5].

In recent years, with the development of morphological analysis techniques and continuous improvements in cell recognition and cell number counting, morphological techniques such as hematoxylin and eosin (HE) staining, immunohistochemical (IHC) staining, immunofluorescence staining, and cell-specific staining have been commonly used in forensic identification. Traditionally, IHC slides are directly extracted from microscopic images using the human eye to obtain qualitative or semi-quantitative experimental results [6], but it is possible to characterize the images artificially. However, artificial observation is subjective, inefficient, and has low reproducibility.

In this study, we introduced an approach to automatically recognize neutrophils in IHC images by establishing cut-off value of the staining area using the TissueFAXS 200 digital pathological analysis system (TissueGnostics GmbH, Vienna, Austria). Compared with artificial recognition, this approach can overcome artificial factors and achieve more objective conclusions. Standardization of the counting method is crucial, and an automatic method is more efficient and accurate. However, an optimal cut-off value of the staining area has not yet been determined using the TissueFAXS 200 digital pathological analysis system in forensic medicine, which may negatively affect clinicians and decrease test accuracy. The purpose of this study was to train, validate, and test the diagnostic effects of the TissueFAXS 200 digital pathological analysis system for neutrophils and compare the results with those of manual counting. We determined the optimal cut-off value of the staining area based on sensitivity, specificity, and receiver operating characteristic (ROC) curve analysis, and rapidly and accurately identified neutrophils during the regeneration of contused skeletal muscle.

## Materials and methods

**Figure 1.**
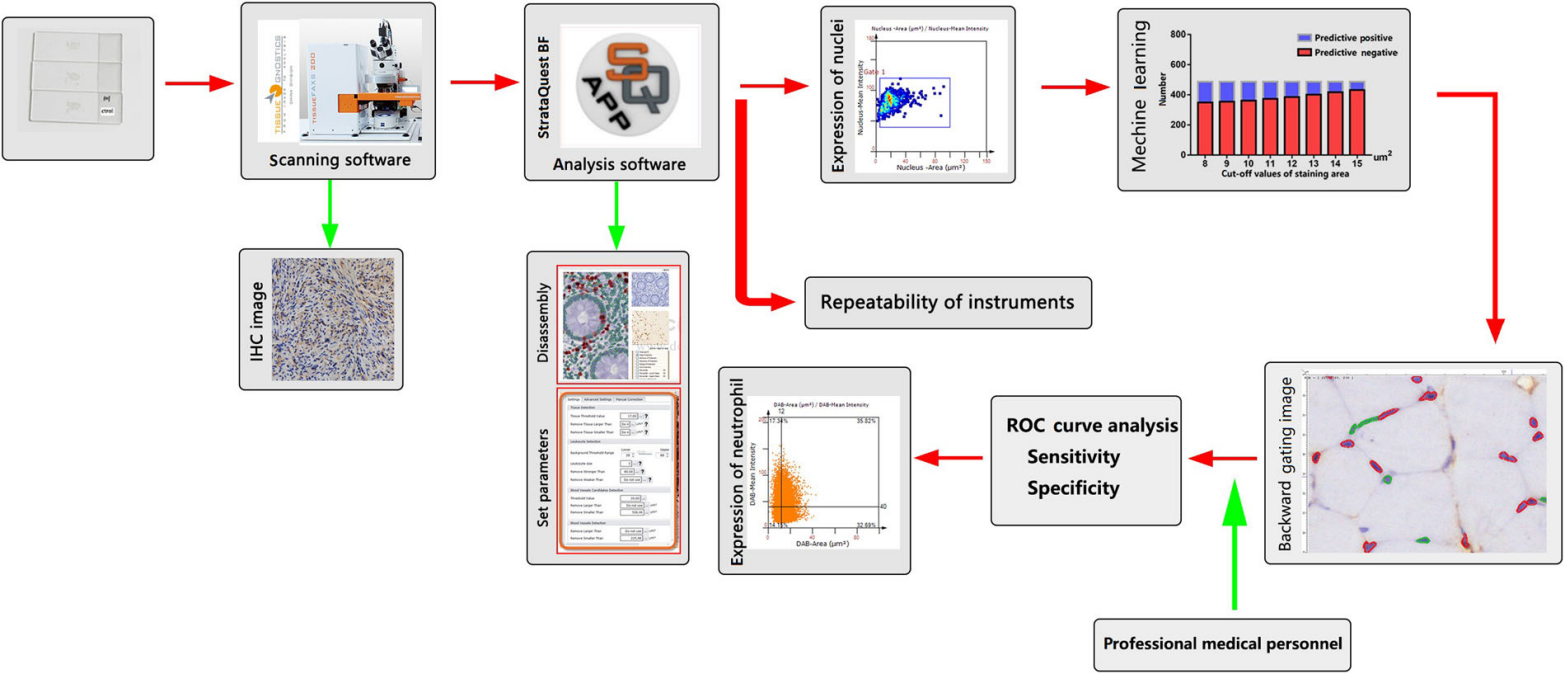
Experimental design for the study. To identify neutrophils during the regeneration of contused skeletal muscle, we introduced an approach to automatically recognize nuclei in immunohistochemical (IHC) images by establishing cut-off value of staining area using the TissueFAXS 200 digital pathological analysis system. Neutrophils were then identified in different “staining areas”, and the optimal staining area was determined based on receiver operating characteristic (ROC) curve analysis. The ratio of neutrophils may be a useful marker for wound aging.

To identify neutrophils during the regeneration of contused skeletal muscle, we introduced an approach to automatically recognize nuclei in IHC images by establishing cut-off value of staining area using the TissueFAXS 200 digital pathological analysis system. Neutrophils were then identified in different staining area, and the optimal cut-off value of the staining area was determined using ROC curve analysis. Finally, the ratio of neutrophils may be a useful marker for wound aging. The experimental design for the study is shown in Figure 1.

### Animal model of skeletal muscle contusion

A total of 55 male Sprague–Dawley (SD) rats (age, 10–12 weeks old; weight, 180–220 g) were purchased from the Animal Center of Shanxi Medical University. All animals were housed in cages with rat chow and water under a 12-h light-dark cycle at 22 °C–24 °C and a relative humidity of 40%–60%.

The 55 healthy adult male SD rats were randomly divided into a control group and contusion groups at 1, 1.5, 2, 3, 4, and 6 h, as well as 1, 3, 5, and 15 d post-injury (*n* = 5 per group). An animal model of skeletal muscle contusion was described previously [7,8]. Briefly, after the rats were anesthetized with pentobarbital sodium, the hair on their right posterior limbs was removed using a depilatory agent (Nair; Carter Wallace, New York, NY, USA). Subsequently, the rats were placed on a foam bed and a 250 g counterpoise was allowed to fall freely 150 cm through a clear Lucite guide tube onto the right posterior limb of the rats [9,10]. After the injury, rats were allowed to recover from the anesthetic and were housed in a cage and fed commercial rat food and tap water *ad libitum*. The rats were anesthetized at the corresponding time points after injury. After heart perfusion with normal saline, rats were sacrificed and skeletal muscle from the right posterior limb was dissected. Rats in the control group did not receive contusion, after which the experimental protocol was identical to that used in the experimental group.

### IHC staining

Immunohistochemistry was performed as described previously [11]. Skeletal muscle was fixed with 4% paraformaldehyde and embedded in paraffin, and 5-µm-thick specimens were mounted on slides, deparaffinized, and rehydrated in a series of graded alcohol solutions. This was followed by antigen retrieval in citrate buffer (pH 6.0; 0.1 mol/L). To quench the endogenous peroxidase activity, sections were further incubated for 10 min in 3% H_2_O_2_. Non-specific binding was blocked by incubation with 5% bovine serum in BSA for 30 min. The blocked sections were incubated overnight at 4 °C with rabbit anti-MPO polyclonal antibody (1:50, ab9535; Abcam, Cambridge, MA, USA) and then rinsed and incubated with an SA1020 mouse/rabbit IgG immunohistochemical staining kit (Boster Biological Technology, Wuhan, China) and secondary antibodies for 20 min at room temperature. The slides were exposed to the colorimetric reagent 3,3-diaminobenzidine tetrahydrochloride (DAB) for 5 min, counterstained for 2 min with Mayer’s hematoxylin, and mounted for evaluation.

### Data acquisition using TissueFAXS software

High-quality images were obtained with TissueFAXS 200 digital pathological analysis system. Data analysis was performed using StrataQuest software (TissueGnostics) [12]. The software programs are based on single cell detection by identification of nuclear structures [13,14]. Nuclei and neutrophils were identified using the “disassembly” function. Furthermore, the average nuclear size, discrimination area, discrimination gray, and background threshold for the master marker was specified. All images were analyzed with the same settings after adjustments. Forward and backward gating was routinely used for quality control. Backward gating was used to verify data by visual inspection on the original image. The results are visualized on dot plot scatter grams and/or histograms. Cut-offs (to differentiate between neutrophils and no-neutrophils) and gates (nuclei and no-nuclei) were set in the scatter grams.

### Determination of neutrophil number

After automatic recognition of hematoxylin and DAB staining, nuclei and neutrophils were identified through the “staining area” and “mean intensity” cut-off value parameters in StrataQuest software. In this report, a total of 20 injury site areas of 0.25 mm^2^ (0.5 mm × 0.5 mm) were randomly selected at all time points to detect the numbers of nuclei labeled with hematoxylin (Hematoxylin) and the percentages of neutrophils labeled with myeloperoxidase (Myeloperoxidase, MPO).

**Figure 2.**
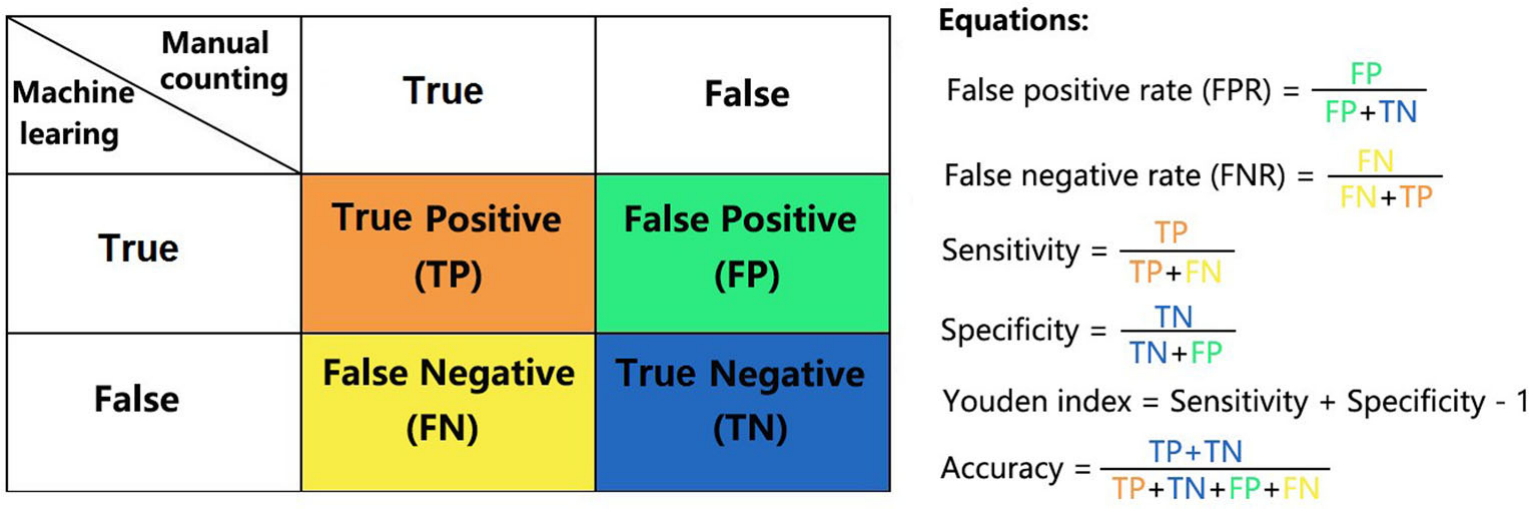
Confusion matrix and performance equations. The confusion matrix included four measurements, as follows: True Positive (TP): standard is positive and predicted is positive. True Negative (TN): standard is negative and predicted is negative. False Positive (FP): standard is negative and predicted is positive. False Negative (FN): standard is positive and predicted is negative. From this confusion matrix, we described a number of overall performance measures: False positive rate (FPR): ratio of false positives predicted (specificity is 1 − FPR); False negative rate (FNR): ratio of false negatives predicted (sensitivity is 1 − FNR); Sensitivity: ratio of positives correctly predicted; Specificity: ratio of negatives correctly predicted; Youden index: evaluates the authenticity of screening tests; Accuracy: overall predictive accuracy of the model.

Intensity was fixed and neutrophils were identified based on the cut-off value of the staining area. The numbers of neutrophils and no-neutrophils under the cut-off value of the staining area were detected using the TissueFAXS 200 digital pathological analysis system. Neutrophils were observed by five professional medical personnel, whose medical knowledge was used as the gold standard. The numbers of false positives (FP) and false negatives (FN) under different cut-off values of the staining area were detected by comparing the numbers of neutrophils identified by TissueFAXS with that observed by professional medical personnel. We observed IHC slides and prepared a confusion matrix (Figure 2).

### Statistical analysis

SPSS 24.0 software (IBM Corp., Armonk, NY, USA) was used for statistical analysis. The predicted positive numbers and predicted negative numbers under different cut-off values of the staining area were calculated. The false positive rate (FPR), false negative rate (FNR), sensitivity, specificity, Youden index, and accuracy were respectively calculated under cut-off values of the staining area, and the authenticity and reliability were determined. Results are expressed as the mean ± standard deviation (SD). *P*-values < 0.05 were considered significant. The test was performed using ROC curve analysis and the test level was area under the curve (AUC) of ROC = 0.7.

## Results

### General condition of skeletal muscle tissue

**Figure 3.**
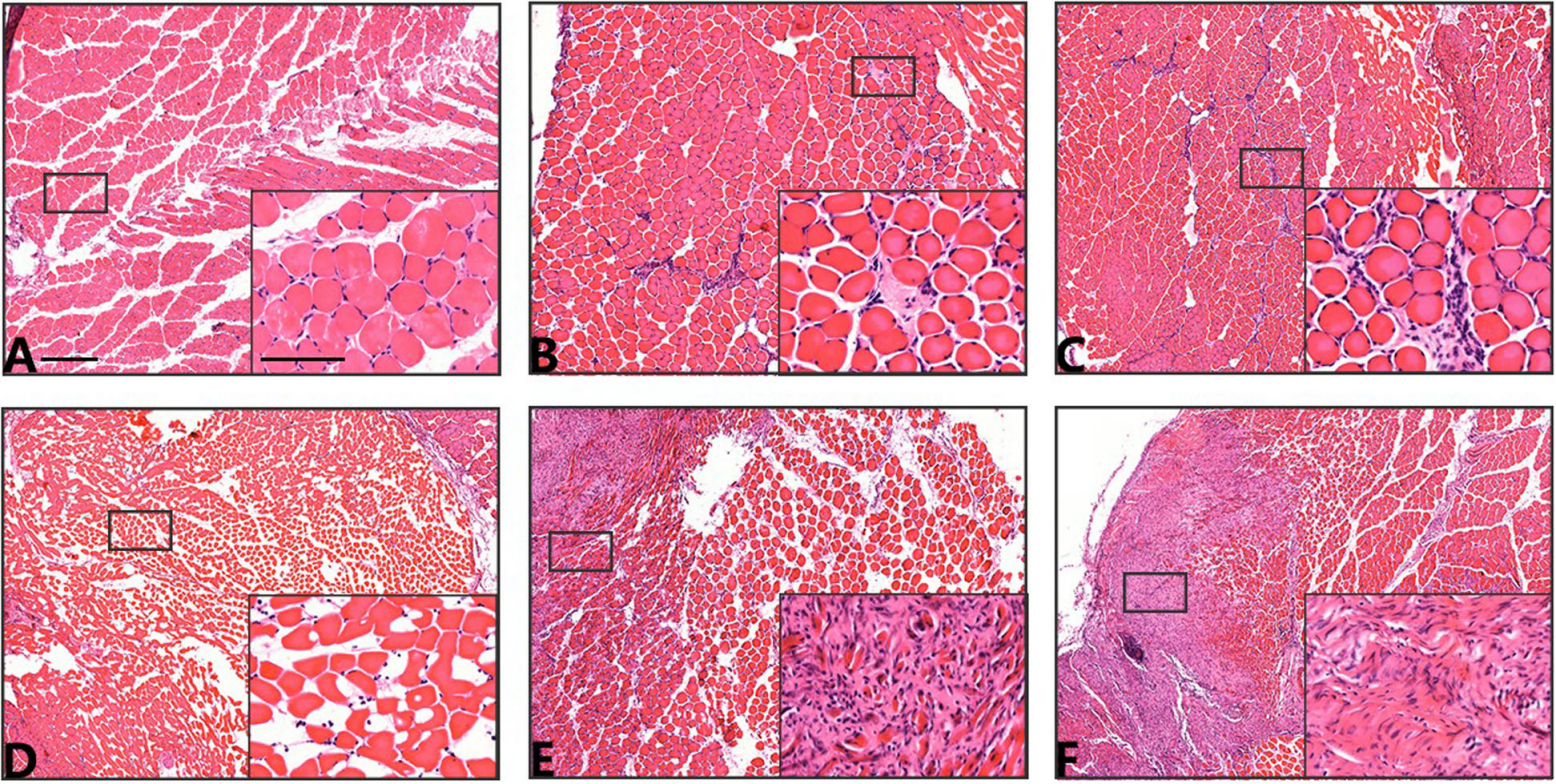
Hematoxylin and eosin staining of skeletal muscle in the post-contusion and control groups. Representative images are shown (scale bar: 500 µm). Insets are higher magnifications (scale bar: 50 µm). (A) Morphology of normal skeletal muscles as a control. (B) Two hours after injury: muscle cells are disordered, cells are swollen, nuclear pyknosis has occurred, and a small number of inflammatory cells have infiltrated. (C) Four hours after injury: muscle cells have degenerated and necrotized, some nuclei are ruptured, and a large number of inflammatory cells have infiltrated. (D) One day after injury: connections between muscle cells are broken, necrotic tissue (muscle cells) has been removed, and the nuclei are broken and dissolved. (E) Three days after injury: skeletal muscle is regenerating and the number of inflammatory cells has decreased significantly. (F) Fifteen days after injury: fibrous connective tissue has proliferated, and the number of nuclei has increased significantly.

In the control group, skeletal muscle tissue was arranged densely in HE staining. The muscle cells were multinucleated, nuclei were located under the cell membrane, and cytoplasm was brightly stained. Compared with the control group, within 1 day after injury the tissue was loosened, the skeletal muscle cells were swollen and disordered, the cells were broken. In addition, pyknosis, karyorrhexis and karyolysis had occurred and inflammatory cells had infiltrated the cytoplasm and surrounding cells. The majority of myocytes dissolved and disappeared within 3 to 15 d after injury and were replaced by fibrous connective tissue. Some muscle cells had dissolved, infiltrating inflammatory cells were still visible, and skeletal muscle began to regenerate (Figure 3).

### Repeatability of the TissueFAXS 200 digital pathological analysis system

To test the repeatability of the TissueFAXS 200 digital pathological analysis system, a total of 20 injury site areas of 0.25 mm^2^ (0.5 mm × 0.5 mm) (*n* = 20) was randomized and the test was repeated 9 times. A replicate test gave similar results with a coefficient of variation (CV) of 0%, indicating that the instruments have good reproducibility and stability and the test can accurately reflect wound aging.

### Determination of nuclei

High-quality images were obtained with the TissueFAXS 200 digital pathological analysis system. After automatic recognition of hematoxylin and DAB staining, the nuclei and neutrophils were identified through the staining area and mean intensity under different cut-off values of staining area in StrataQuest software.

**Figure 4.**
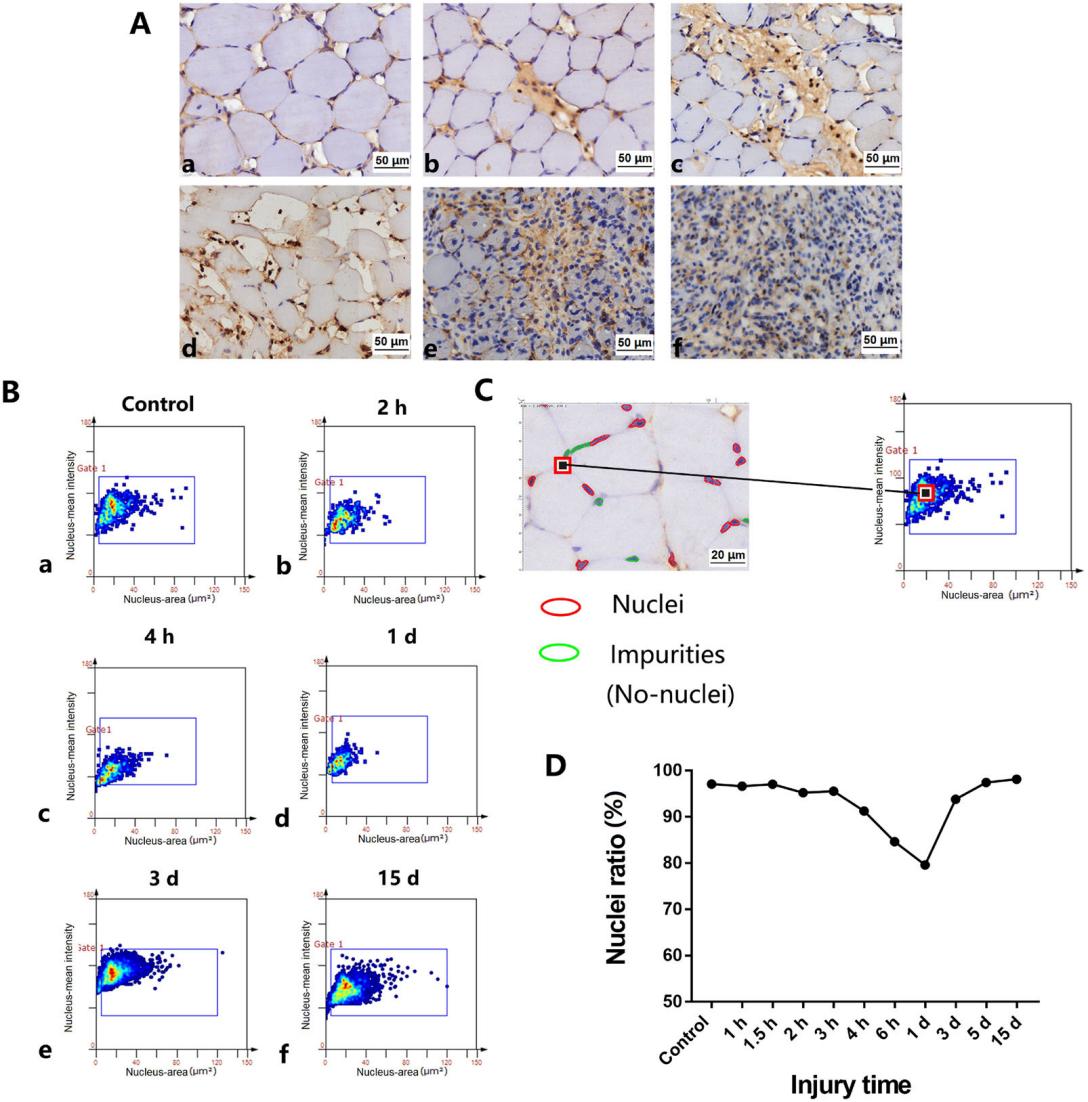
Detection and quantification of nuclei in rat skeletal muscle cells at different time points post-injury. (A) Immunohistochemistry of nuclear expression at different time points. (a) Morphology of normal skeletal muscles as a control. (b–f) Nuclei are present in the injured areas at 2 h, 4 h, 1 d, 3 d, and 15 d after contusion, respectively. Scale bar: 50 µm. (B) Scatterplot of nuclear expression at different time points after injury. (C) Scatterplot of the analyzed IHC samples used in panel (A). Discrimination of nuclei to the control group nuclei. Image of gated (nuclei, red circles) and no-nuclei or impurities (green circles) in an IHC-stained skeletal muscle sample. Scale bar: 20 µm. (D) Expression of nuclei at different time points after injury. The nuclei ratio refers to the ratio of nuclei to the total numbers of nuclei and impurities. The average ratio of hematoxylin-positive nuclei were minimal at 1 d post-injury.

The IHC results of hematoxylin-labeled nuclei were as follows (Figure 4A). After skeletal muscle contusion, the numbers of nuclei were regulated to a certain degree (Figure 4D). In the control group, because of impurities caused by the dye, the ratio of nuclei to the total numbers of nuclei and impurities was 97.13%. The muscle fiber structure of the damaged tissue was destroyed within 1 d after injury, the neutrophils phagocytosed necrotic tissues, pyknosis, karyorrhexis, and karyolysis had occurred, and the proportions of nuclei in the skeletal muscle decreased. Regeneration and repair began 3 d after injury, and the ratio of nuclei in skeletal muscle increased significantly. After automated nuclear detection, nuclear size in the control group was used to discriminate between the nuclei and no-nuclei (Figure 4C, left). Forward and backward gating was routinely used for quality control. By clicking on a dot of the scatterplot, the forward gating showed the staining area and mean intensity of selected nuclei in the scatter gram. Backward gating was used to verify data by visual inspection on the original image (Figure 4C, right).

### Expression of neutrophils under different cut-off values

Neutrophils play an important role in the early stages of inflammation after tissue injuries and for wound age estimates. Due to the lack of quantification and standardization at each step of IHC, the intensity may vary. Therefore, the cut-off value of mean intensity could not be directly compared between different slides. To quickly and accurately identify the numbers of neutrophils, we determined the optimal cut-off value of the staining area.

**Figure 5.**
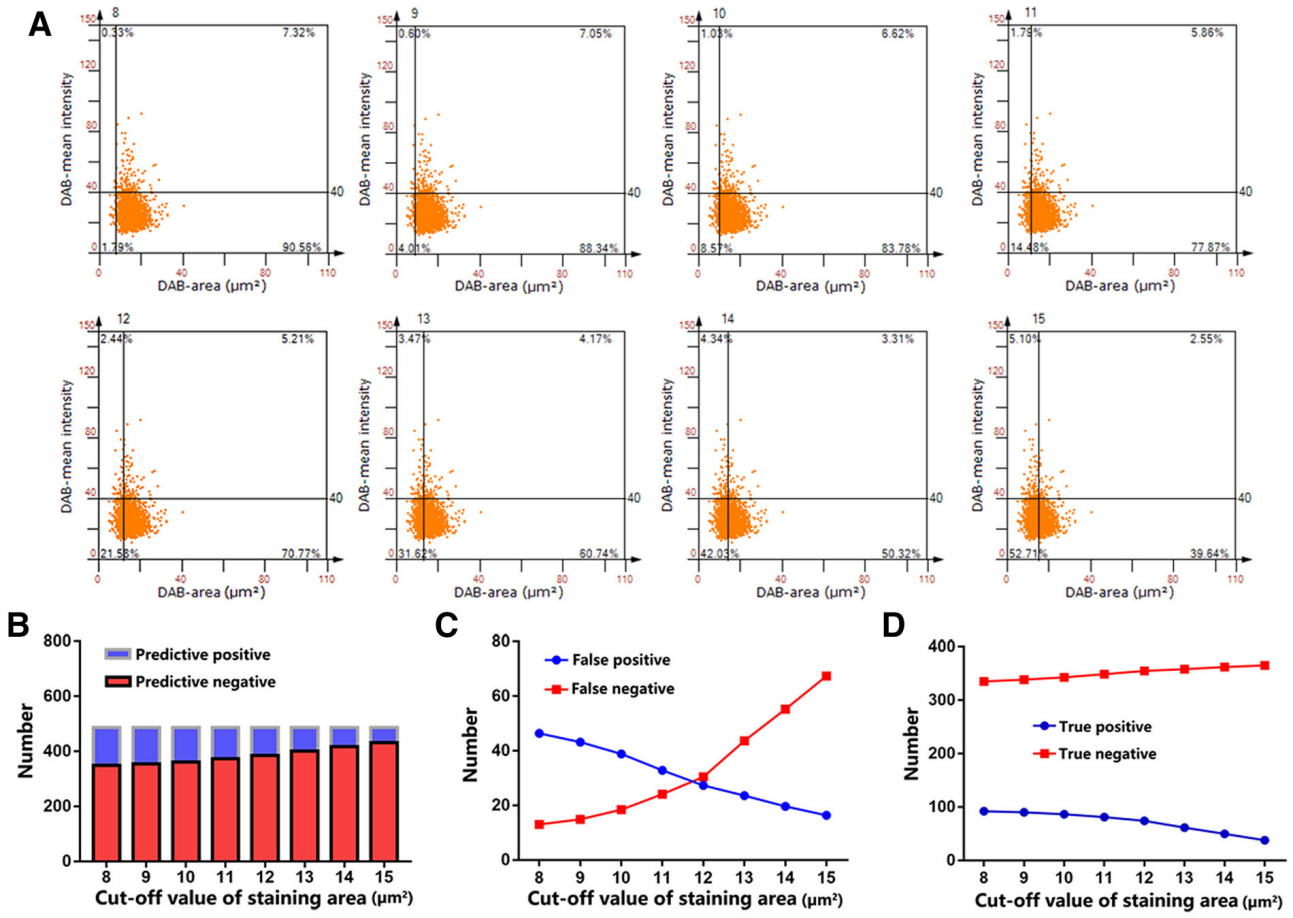
Changes in neutrophils under different cut-off values of staining area. (A) Scatterplot of changes in neutrophil numbers at different cut-off values of staining area. (B) In the same region, the numbers of predicted positives decreases and the numbers of predicted negatives increases as cut-off values of the staining area increase. (C) As the cut-off value of staining area increases, the numbers of false positives observed decreased and those of false negatives increased. (D) As the cut-off value of staining area increases, the numbers of true positive decreased and those of true negative increased.

As shown in Figure 5B, we determined the numbers and proportions of neutrophils under different cut-off values of the staining area. The results showed that as the cut-off value of staining area increased, the predicted positive numbers (neutrophils) decreased, and the predicted negative numbers increased (Figure 5A,B). We expected that more neutrophils would be detected as no-neutrophils with higher cut-off values of the staining area. Similarly, as the cut-off value of staining area increased, the numbers of FP decreased, and the numbers of FN increased (Figure 5C). As the cut-off value of staining area increased, the numbers of TP decreased, and the numbers of TN increased (Figure 5D). Specific screening indicators of different cut-off values staining area are shown in Supplementary Table 1.

### Accuracy of the TissueFAXS 200 digital pathological analysis system

To evaluate the identification of neutrophils and explore the accuracy of the TissueFAXS 200 digital pathological analysis system, we compared the results of machine learning with manual counting under a microscope. According to the gold standard of neutrophils observed by professionals, the numbers of FP, FN, TN, and TP under the different cut-off values of staining area were obtained (Supplementary Table 1). All neutrophils were measured under different cut-off values of staining area. The FPR ranged from 4.28% to 12.14%. The FNR ranged from 12.42% to 64.08%. The sensitivity was 35.92%–87.58% and the specificity was 87.86%–95.72%. The Youden index was 0.316–0.754, and the accuracy was 82.80%–88.30% (Table 1).

**Table 1.** Comparison of the authenticity and reliability of different cut-off values of staining area.

Staining area of neutrophils (μm²)	FPR (%)	FNR (%)	Sensitivity (%)	Specificity (%)	Youden index	Accuracy (%)
8	12.14	12.42	87.58	87.86	0.754	87.80
9	11.31	14.18	85.82	88.69	0.745	88.07
10	10.17	17.55	82.45	89.83	0.723	88.24
11	8.61	22.93	77.07	91.39	0.685	88.30
12	7.15	29.08	70.92	92.85	0.638	88.14
13	6.17	41.53	58.47	93.83	0.523	86.19
14	5.15	52.57	47.43	94.85	0.423	84.61
15	4.28	64.08	35.92	95.72	0.316	82.80

FPR: false positive rate; FNR: false negative rate. Youden index = Sensitivity + Specificity − 1.

**Figure 6.**
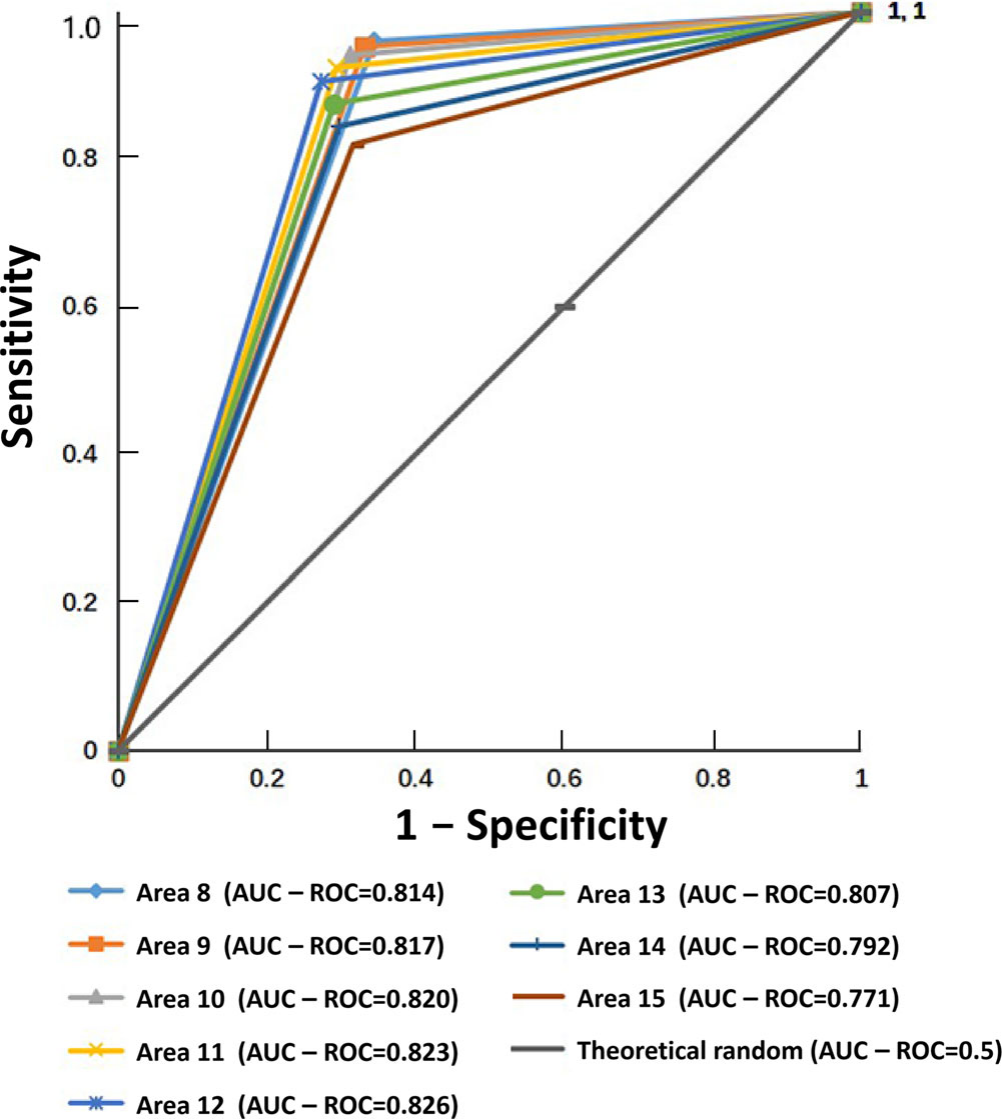
Receiver operating characteristic (ROC) curves of different out-off values of staining area (8–15 µm^2^). Sensitivity is taken as the ordinate to represent the true positive rate, (1 − specificity) as the abscissa represents the false positive rate; the plot is plotted as the ROC curve. The dark gray line is the line of no discrimination derived from a theoretically random classification. Data are presented as area under the ROC curve and analyzed using ROC curves, and the test level is area under the curve (AUC) = 0.7.

We used ROC curves to determine the performance of the instrument (Figure 6). This curve is used to describe the trade-off between the TPR and the FPR of the method. The AUC of ROC curve and standard errors were 0.814(±0.01), 0.817(±0.01), 0.820(±0.01), 0.823(±0.01), 0.826(±0.01), 0.807(±0.01), 0.792(±0.01), and 0.771(±0.01) under cut-off values of staining area of 8–15 µm^2^, respectively. The AUC was largest when the cut-off value of the staining area was 12 µm^2^, indicating that the instrument has good reliability and good performance.

### Relative expression of neutrophils at different injury times

**Figure 7.**
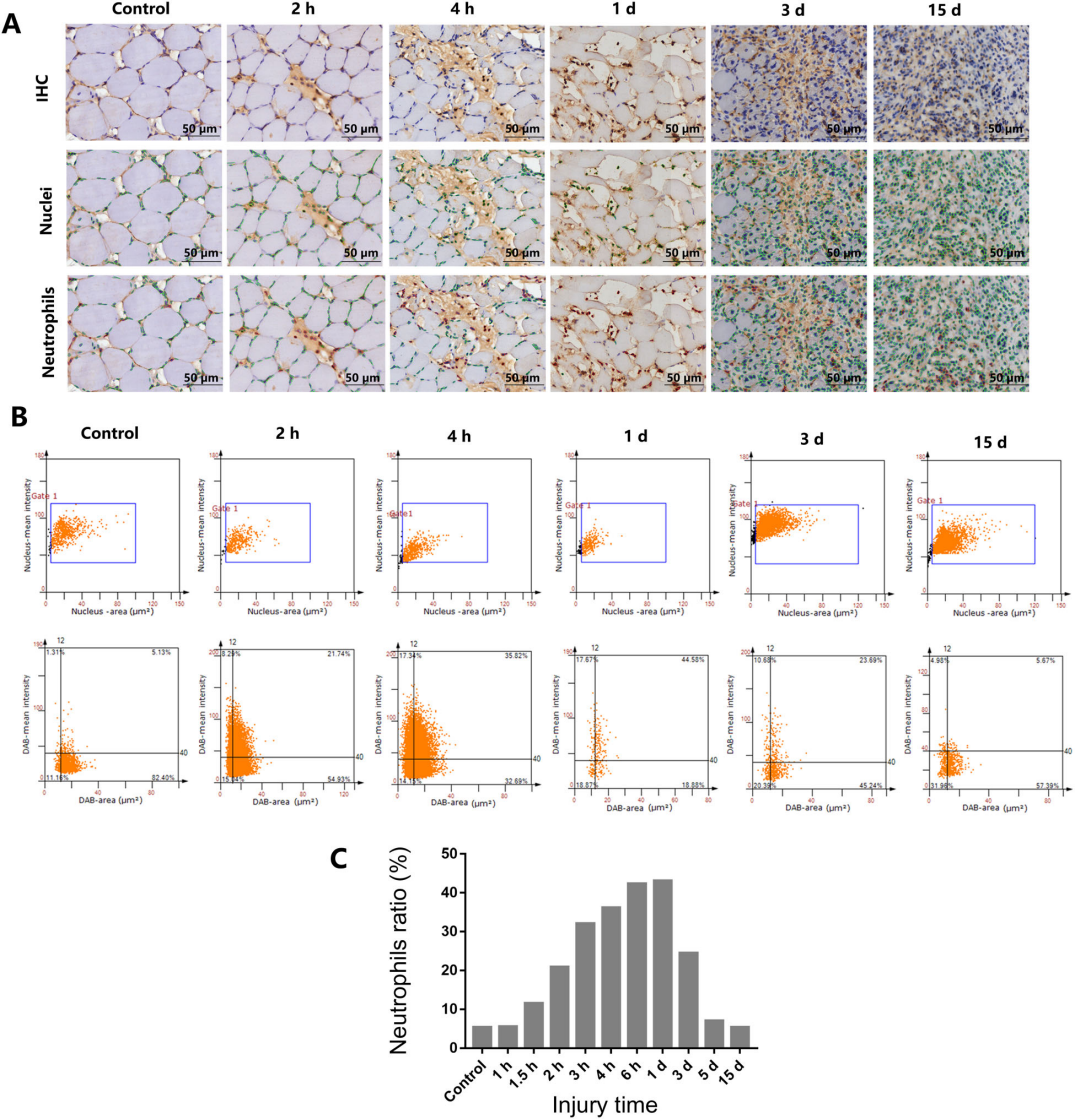
Detection and quantification of neutrophils in rat skeletal muscle cells at different time points after injury. (A) IHC of nuclei and neutrophil expression at different injury times and in control rats. Scale bar: 50 µm. Nuclei (green circles) and neutrophils (red circles) present in the injured areas at 2 h, 4 h, 1 d, 3 d, and 15 d after contusion. (B) Scatterplot of nuclei and neutrophils expression at different injury times and in control rats. (C) Expression of neutrophils at different time points after injury. The average ratio of MPO-positive neutrophils to the total numbers of nuclei were calculated for each injured area, and is peaked at 1 d post-injury.

When the cut-off value of the staining area was 12 µm^2^, the numbers and percentages of neutrophils in IHC images were detected using the TissueFAXS 200 digital pathological analysis system (Figure 7). The average ratios of MPO-positive neutrophils to the total numbers of nuclei were calculated for each injured area, and the results were obtained directly from the class flow graph. As shown in Figure 7, the proportions of neutrophils changed regularly after skeletal muscle contusion. Compared with the control group, neutrophils began to increase at 1.5 h after injury. Subsequently, neutrophils migrated to the injury site and phagocytosed the necrotic tissue, where pyknosis, karyorrhexis, and karyolysis had occurred. In addition, the proportion of neutrophils increased and peaked 1 d after injury. Regeneration and repair began 3 d after injury and the numbers of nuclei in skeletal muscle increased significantly, whereas the proportion of neutrophils decreased.

## Discussion

Skeletal muscle injury is one of the most common injuries in forensic science [15]. After acute skeletal muscle injury, the injury site experiences damage, degeneration, and necrosis of the muscle fiber structure, after which inflammatory cells and factors infiltrate the injury site [16–20]. Neutrophils play important roles in the early stages of inflammation after various tissue injuries. Neutrophils rapidly infiltrate the injured area to phagocytose and remove necrotic tissue under chemotaxis by inflammatory factors [21], which creates conditions for tissue repair and regeneration in the damaged areas.

The TissueFAXS 200 digital pathological analysis system is the world’s newest system integrating immunohistochemistry, immunofluorescence, and *in situ* quantitative analysis of panoramic images. The system enables panoramic imaging of tissues and cells, as well as *in situ* morphological analysis of tissues and cells. It has a wide range of applications, including tumour cell identification, neural cell differentiation, organ transplantation, drug screening, cyclin, pharmacodynamic analysis, cancer research, developmental biology, metabolic physiology, immunology, dermatology, urology, drug development, and clinical diagnosis.

The study of neutrophils in IHC images showed that the inefficient and subjective artificial recognition using traditional methods influences the accuracy of experimental results. At this time, image analysis methods based on optical density and mean gray values were used [22,23]. Light density reflects the intensity of proteins in positive cells; however, due to a lack of quantification and standardization at each step of IHC, many factors [24,25], including the developing time of DAB, antigen recovery (or antigen repair) and antigen exposure, temperature, and cleanliness of the slides influence the accuracy of the experimental results and increase the FPR, which is the greatest challenge in image analysis methods. Therefore, in this study, the nuclei were identified based on background correction. The major changes in nuclei during injury repair included pyknosis, karyorrhexis, and karyolysis [26–28]. Furthermore, the instrument has limitations, and morphologically changed nuclei were recognized as normal nuclei, which increased the FPR. Therefore, it is important to precisely recognize neutrophils using the sensitivity and specificity of the instrument. Furthermore, we can use this instrument to perform statistical analyses on different batches of immunohistochemical slides.

At this time, forensic identification applications of this technology still require improvement. Thus, we need to improve the performance of the instrument and select the optimal cut-off value of the staining area. The TissueFAXS 200 digital pathological analysis system was shown to have good reproducibility and accuracy based on the reproducibility test, specificity and sensitivity, and ROC curve analysis. As the neutrophil staining area increased, the sensitivity decreased, specificity increased, and accuracy showed a specific trend.

In addition, the optimal cut-off value of the neutrophil staining area was determined using ROC curve analysis. The ROC curve is a graphical representation of the relationship between the sensitivity and specificity of a diagnostic test, which describes the relationship between the sensitivity and specificity of an analytical method and is a comprehensive representation of test accuracy. In this experiment, the AUC was largest when the neutrophil staining area was 12 µm^2^, indicating that the performance of the instrument was good. This approach overcomes artificial factors and achieves more objective conclusions, and an automatic method saves time and has higher accuracy. Determining the optimal cut-off value of the staining area is important to increase sensitivity and reduce specificity. Specifically, it not only effectively improves the sensitivity of the TissueFAXS 200 digital pathological analysis system, but also reduces the related workload of clinical staff and provides conditions for further research. The TissueFAXS 200 digital pathological analysis system can also be used to optimize the conditions of different cell types under various injury conditions to determine the optimal cut-off value of the staining area and provide optimal conditions for further study.

The inflammatory response is triggered after acute muscle contusion. The body eliminates and absorbs necrotic tissue and cells and repairs the damage to achieve self-defense and protection [29,30]. Previous studies have shown that neutrophils exhibit time-dependent expression after skeletal muscle injury in rats [4,31]. The results have shown that the numbers of neutrophil infiltration is obvious 6 to 12 h after skeletal muscle contusion, after which the degree of infiltration begins to decrease 1 d after injury, clearly decreases 3 d after injury, and then disappears [4].

In present study, the neutrophils ratio, which refers to the ratio of neutrophils to the total numbers of nuclei, was firstly introduced in wound age estimation as an important indicator. As showed in Figure 7C, the ratio of neutrophils began to increase at 1.5 h after injury, and the proportion of neutrophils peaked at 1 d after injury and decreased sharply at 3 d after injury. Compared with Guan’ opinions [4], it is a new discovery that the proportion of neutrophils peaked at 1 d after injury. It raised new insights on the expression of neutrophils during injury time. Moreover, in this study, we found that the numbers of nuclei began to decrease transiently due to pyknosis, karyorrhexis, and karyolysis in the early stages of muscle injury. Subsequently, the numbers of nuclei consistently increased because of neutrophil infiltration and proliferation and differentiation of muscle satellite cells.

In the future, it is very important to have a more comprehensive evaluation in wound age estimation and the research should combine the counting of neutrophils with other inflammatory cells. So, it is necessary to identify various cell types by establishing cut-off values based on the TissueFAXS 200 digital pathological analysis system. Moreover, it is true that the standardization of the counting method is crucial, and an automatic method is timesaving and more accurate. With the popularization of image analysis technology and continuous functional improvements, it will gradually be integrated with clinical practice and become a powerful tool for pathological diagnosis.

## Conclusion

In conclusion, investigation of the time-dependent expression of neutrophils could help determine the age of early wounds. Based on sensitivity, specificity, and ROC curve analysis, neutrophils can be accurately identified during regeneration of contused skeletal muscle. Substituting machine learning for manual counting increases the accuracy and efficiency of counting devices, overcomes artificial factors, and achieves more objective conclusions, facilitating research into the estimation of wound age.

## Supplementary Material

Supplemental Material
