# Supplementary material for: Analysis of sensitivity and specificity: precise recognition of neutrophils during regeneration of contused skeletal muscle in rats
Source: Forensic Sci Res. 2020 Mar 19;7(2):228–37. doi: 10.1080/20961790.2020.1713432 (PMC9245985; doi:10.1080/20961790.2020.1713432)
Supplement: Supplemental Material [file TFSR_A_1713432_SM6178.docx]

**Supplementary Table 1. Comparison of screening indicators at different cut-off values of staining area.**

|  | Machine Learning | |  | Manual Counting | | | |
| --- | --- | --- | --- | --- | --- | --- | --- |
|  | Predictive  positive | Predictive  negative |  | False  positive | False  negative | True  positive | True  negative |
| area8 | 138.40±81.93 | 348.35±131.40 |  | 46.35±26.85 | 13.05±8.03 | 92.05±61.64 | 335.30±131.26 |
| area9 | 133.35±79.22 | 353.40±130.02 |  | 43.15±25.01 | 14.90±8.28 | 90.20±60.41 | 338.50±130.33 |
| area10 | 125.45±75.46 | 361.30±128.01 |  | 38.80±22.24 | 18.45±10.64 | 86.65±58.86 | 342.85±128.80 |
| area11 | 113.85±69.22 | 372.90±124.39 |  | 32.85±18.37 | 24.10±12.40 | 81.00±55.24 | 348.80±126.93 |
| area12 | 101.55±62.75 | 385.20±120.92 |  | 27.30±14.39 | 30.45±15.88 | 74.25±52.13 | 354.75±127.56 |
| area13 | 85.00±54.54 | 401.75±115.70 |  | 23.55±12.73 | 43.65±22.93 | 61.45±45.80 | 358.10±124.15 |
| area14 | 69.50±45.12 | 417.25±110.70 |  | 19.65±11.21 | 55.25±30.92 | 49.85±38.35 | 362.00±122.27 |
| area15 | 54.10±36.42 | 432.65±106.55 |  | 16.35±8.83 | 67.35±37.14 | 37.75±31.19 | 365.30±121.45 |
